# LKB1/AMPK inhibits TGF-β1 production and the TGF-β signaling pathway in breast cancer cells

**DOI:** 10.1007/s13277-015-4639-9

**Published:** 2015-12-30

**Authors:** Nian-Shuang Li, Jun-Rong Zou, Hui Lin, Rong Ke, Xiao-Ling He, Lu Xiao, Deqiang Huang, Lingyu Luo, Nonghua Lv, Zhijun Luo

**Affiliations:** 1 Research Institute of Digestive Diseases and Department of Gastroenterology, The First Affiliated Hospital, Nanchang University, Nanchang, Jiangxi China; 2 Graduate Program of Basic Medical Sciences, School of Basic Medical Sciences, Nanchang University, Nanchang, Jiangxi China; 3 Department of Biochemistry, Boston University School of Medicine, 72 East Concord Street, Boston, MA 02118 USA

**Keywords:** LKB1, AMPK, TGF-β production and signaling, Breast cancer cell migration, Epithelial-to-mesenchymal transition

## Abstract

Adenosine monophosphate-activated protein kinase (AMPK) acts as a fuel gauge that maintains energy homeostasis in both normal and cancerous cells, and has emerged as a tumor suppressor. The present study aims to delineate the functional relationship between AMPK and transforming growth factor beta (TGF-β). Our results showed that expression of liver kinase B1 (LKB1), an upstream kinase of AMPK, impeded TGF-β-induced Smad phosphorylation and their transcriptional activity in breast cancer cells, whereas knockdown of LKB1 or AMPKα1 subunit by short hairpin RNA (shRNA) enhanced the effect of TGF-β. Furthermore, AMPK activation reduced the promoter activity of TGF-β1. In accordance, type 2 diabetic patients taking metformin displayed a trend of reduction of serum TGF-β1, as compared with those without metformin. A significant reduction of serum TGF-β1 was found in mice after treatment with metformin. These results suggest that AMPK inhibits the transcription of TGF-β1, leading to reduction of its concentration in serum. Finally, metformin suppressed epithelial-to-mesenchymal transition of mammary epithelial cells. Taken together, our study demonstrates that AMPK exerts multiple actions on TGF-β signaling and supports that AMPK can serve as a therapeutic drug target for breast cancer.

## Introduction

Adenosine monophosphate-activated protein kinase (AMPK) acts as a fuel-sensing enzyme that plays an important role in regulating energy metabolism in both normal and malignant cells. Furthermore, it has emerged as a tumor suppressor that mediates the tumor-suppressive function of liver kinase B1 (LKB1) [1, 2]. A plethora of studies have demonstrated that AMPK regulates a broad spectrum of factors involved in cell metabolism, proliferation, survival, migration, and invasion [3]. Interestingly, a seminal retrospective investigation has reported that the incidence of cancer is significantly reduced in patients with type 2 diabetes receiving metformin, an AMPK activator, as a glucose-lowering drug [4]. Afterwards, several clinical studies have demonstrated that expression of LKB1 or AMPK activity is reduced in advanced breast cancer [5, 6]. The alteration is associated with histological grades, metastasis, and poor prognosis. Intriguingly, studies have compared the complete pathological response or distant metastasis in diabetic patients complicated with breast cancer who received metformin in neoadjuvant chemotherapy to those without it [7]. The results have shown that metformin causes a higher complete pathological response rate and that the patients not taking metformin have a trend to distant metastasis. Therefore, these clinical studies suggest that AMPK may be a therapeutic target for breast cancer progression.

The transforming growth factor beta (TGF-β) family regulates many aspects of cellular functions including cell growth, differentiation, adhesion, migration, and apoptosis, which are involved in both physiological and pathophysiological processes [8]. While TGF-β exerts a suppressive effect in early stages of tumorigenesis, hence regarded as a tumor suppressor, it promotes tumor progression and metastasis in late stages [8]. In fact, many studies have shown that serum levels of TGF-β increase in breast cancer and other cancers and can serve as a predictive and prognostic marker of cancer stage [8–11]. Thus, persistently high circulating TGF-β levels after curative removal of primary tumor predicts early metastatic recurrence in distant organs while its decrease correlates with response to treatment [12]. Increased secretion of TGF-β from tumors renders them more resistant to chemotherapy, whereas antagonizing TGF-β with neutralizing antibody or inhibitors can increase the chemotherapeutic sensitivity of tumor cells [13–16]. The activation of the TGF-β signaling pathway promotes metastasis of cancer via complex mechanisms, one of which is to regulate epithelial-to-mesenchymal transition (EMT), a critical step for cancer stem cell (CSC) transition and cancer metastasis [17].

In recent years, many studies have shown that AMPK plays an inhibitory role in EMT, tissue fibrosis, and malignant transformation [18–25]. However, mechanisms underlying EMT are complex involving multiple factors and pathways. The negative regulation impinged upon TGF-β constitutes one of the mechanisms. With regards to the effect on TGF-β signaling, it has been reported that this could occur through inhibition of Smad2/3 phosphorylation or a Smad3-dependent but phosphorylation-independent event [26, 27]. In exploring the molecular link between AMPK and progression of breast cancer, the present study investigated the effect of AMPK activation on TGF-β signaling. Our results showed that AMPK activation suppressed TGF-β-induced phosphorylation of Smad2/3 phosphorylation in MDA-MB-231 cells and MCF10A cells. Knockdown of LKB1 and AMPKα1 by short hairpin RNA (shRNA) disrupted acinus formation and enhanced responses of the cells to TGF-β in terms of Smad2/3 phosphorylation and promoter activity. Furthermore, our data showed that metformin reduced serum TGF-β1 levels in type 2 diabetic patients and mice. In keeping with this, metformin inhibited the transcriptional activity of TGF-β1 promoter. Finally, AMPK activation attenuates EMT induced by TGF-β1. Altogether, our results demonstrate that AMPK inhibits TGF-β signaling via multiple mechanisms.

## Materials and methods

### Ethical statement for human serum collection

The study using human serum samples was approved by the Human Study Ethics Committee of The First Affiliated Hospital of Nanchang University (Nanchang, China). Sera were collected from patients with type 2 diabetes who were treated with metformin (10 males and 11 females) or with other glucose-lowering drugs (15 males and 14 females) in the outpatient clinic. The age of patients ranged from 45 to 65 years. Blood samples were obtained under the consent of patients.

### Reagents

Metformin and phenformin were purchased from Sigma-Aldrich (St. Louis, MO, USA); human TGF-β1 and antibodies against β-actin, p-AMPKα Thr172, AMPKα, p-ACC Ser79, ACC, Slug, p-Smad3 Ser423/425, and Smad2/3 were from Cell Signaling Technology (Beverly, MA, USA); ELISA kits for human and mouse TGF-β1, antibodies against E-cadherin, and vimentin were from Abcam (Cambridge, MA, USA); monoclonal antibody against LKB1 was from Santa Cruz Biotechnology (Santa Cruz, CA, USA); growth factor-reduced Matrigel and monoclonal antibody against β-catenin were from BD Biosciences (San Jose, CA, USA); Lipofectamine 2000, 4′,6-diamidino-2-phenylindole (DAPI), fluorescein isothiocyanate (FITC)-conjugated donkey anti-rabbit antibodies were from Life Technologies (Grand Island, NE, USA); Chamber slides was from EMD Millipore (Billerica, MA, USA).

### Animal study

Animal protocol was approved by the Institutional Animal Ethics Committee of The First Hospital of Nanchang University. BALB/c mice (6 weeks old) were provided by the Animal Core Laboratory of Jiangxi Medical College, Nanchang University. The blood (0.2 ml) was collected from the orbital sinus after inhaling anesthetization with isoflurane, and the serum was separated and stored at −80 °C prior to use. Animals were injected with metformin (100 mg/kg/day) i.p. for 1 w and euthanized by carbon dioxide, immediately followed by cardiac injection for blood collection. Sera were used for measurement of TGF-β1.

### Cell culture, transfection, and virus infection

MDA-MB-231 cells were cultured in Dulbecco’s modified Eagle’s medium (DMEM) supplemented with 10 % fetal bovine serum (FBS) and NMuMG cells in DMEM supplemented with 10 % FBS and insulin (10 μg/ml) at 5 % CO_2_ and 37 °C. MCF10A cells were cultured in DMEM/F12 cells supplemented with 5 % horse serum, EGF (20 ng/ml), insulin (10 μg/ml), hydrocortisone (0.5 mg/ml), and cholera toxin (100 ng/ml). All the media were supplemented with penicillin/streptomycin (Pen/Strep) solution.

For transient expression, mammalian expression plasmids containing genes of interest were transfected into appropriate cells using Lipofectamine 2000. Forty-eight hours post-transfection, the cells were treated with agents as indicated in the figure legend and lysed with appropriate solutions.

For virus infection, lentivirus encoding shRNAs for green fluorescent protein (GFP) and LKB1 (gift from Bin Zheng, Massachusetts General Hospital) was packaged in 293 T cells and the virus supernatant was infected into MCF10A cells. Lentivirus for AMPKα1 shRNA was purchased from Sigma-Aldrich and infected into MCF10A cells. Two days after infection, the cells were selected with puromycin (1 μg/ml)

### Three-dimensional cell culture

Analysis of acinar structure was carried out as described previously [28]. Briefly, growth factor-reduced Matrigel was allowed to thaw out on ice and 40 μl was added to each well of chamber slides, which was placed to tissue culture incubator to solidify Matrigel. MCF10A cells were trypsinized, resuspended in DMEM/F12 containing 20 % horse serum, and then centrifuged and resuspended again in assay media (DMEM/F12, insulin [10 μg/ml], hydrocortisone [0.5 μg/ml], cholera toxin [100 ng/ml], 2 % horse serum, Pen/Strep). The cell density was adjusted to 5000 cells/400 μl in the assay media supplemented with 2.5 % Matrigel and 5 ng/ml EGF, and the cells were overlayed on the top of a Matrigel chamber. The medium was changed every 4 days until 10∼14 days.

When cells formed clumps under a light microscope, the chamber slides were fixed with 5 % formalin at room temperature for 30 min, washed with phosphate-buffered saline (PBS), and permeabilized with 0.5 % Triton X-100 in PBS for 5 min, washed again, blocked, and subsequently incubated with first antibody, FITC-conjugated second antibody, and DAPI.

### Immunofluorescent cytochemistry

Immunofluorescent labeling of cells was conducted following the provider’s protocol for FITC-conjugated antibody (Life Technologies, Grand Island, NE, USA). Briefly, the cells were plated onto coverslips pre-coated with 0.01 % polylysine. After treatment, the cells were fixed with 4 % paraformaldehyde for 30 min, permeabilized in PBS-0.3 % Triton X-100 for 5 min, and incubated in a PBS and 1 % bovine serum albumin (BSA) blocking solution for 1 h. The cells were incubated with anti-E-cadherin at 4 °C overnight and stained with FITC-conjugated secondary antibody at room temperature for 1 h. Cell nuclei were counterstained with DAPI. All the images were taken under a fluorescent microscope (Nikon C2).

### Western blot analysis

Cells were stimulated with TGF-β1 (5 ng/ml) and metformin (10 mM). Protein concentration was determined using a Coomassie brilliant blue staining method. An equal amount of protein (20∼25 μg) was separated on SDS-PAGE and transferred to nitrocellulose membranes. The membranes were blocked in 5 % nonfat milk prepared in Tris-buffered saline-0.1 % Tween 20 (TBST) at room temperature for 1 h and then incubated with primary antibody in 2 % BSA-TBST at 4 °C from 2 h to overnight. The membranes were washed and incubated with HRP-conjugated secondary antibody. Immuno-reactive protein was detected using an ECL kit (Bio-Rad, USA).

### Luciferase assay

The promoter sequence of human TGF-β1 was selected according to Kim et al. [29], and a 1.4-kb fragment amplified from genomic DNA was isolated from 293 T cells by PCR using the following primers: forward primer, 5′agctctcgag(*Xho*I)TTA GCA GGG GAG TAA CAT GGA TTT3′, and reverse primer, agctaagctt (*Hind*III)AGG GAG GTG GGA GGG AGA TG3′ The DNA segment was digested with *Xho*I and *Hind*III and then subcloned to the site upstream of firefly luciferase in pGL3 plasmid (Promega, USA). The insert was verified by sequencing analysis.

The plasmid encoding a firefly luciferase reporter where a TGF-β1 promoter or Smad2/3 response element lies upstream was co-transfected with the plasmid encoding Renilla luciferase into cells using Lipofectamine 2000. After 48 h, the cells were treated with TGF-β and/or metformin for 8 h. Luciferase activity was determined using a Dual-Luciferase Assay Kit (Promega, USA) and expressed as a ratio of firefly luciferase to Renilla luciferase reading units.

### Measurement of serum TGF-β1 level

Human and mouse serum TGF-β1 levels were measured using ELISA kits from Abcam (Cambridge, USA), according to the protocols provided by the manufacturer. Serum samples were first activated by acidification and then neutralized and diluted. Thus, the concentration reflected total TGF-β1.

### RNA extraction and real-time PCR

Total RNA was extracted using a kit from Thermo Fisher Scientific (Waltham, MA, USA) following the manufacturer’s instructions. Two micrograms of total RNA was used for cDNA synthesis. Real-time PCR was carried out for detection of Twist and zinc finger E-box binding homeobox 1 (ZEB1) using the SYBR Green PCR Master Mix 2× reagent in 20 μl according to the protocol provided by the manufacturer Applied Biosystems (Foster City, CA, USA). PCR was run in triplicates and repeated for three times. Glyceraldehyde 3-phosphate dehydrogenase (GAPDH) was used as a control to normalize the variability in expression levels. Primer sequences for real-time PCR were the following: Twist forward 5′-TGG ACA GAG ATT CCC AGA GG-3′, reverse 5′- TTC CTG TCA GTG GCT GAT TG; ZEB1 forward 5′-AAG CAG CCA GAG AAG AGC TG-3′, reverse 5′- CCA CAT CAA CAC TGG TCG TC; and GAPDH forward 5′- CAT CTT CCA GGA GCG AGA CC-3′, reverse 5′- CTC GTG GTT CAC ACC CAT C-3′.

### Wound healing assay

Cell monolayers were scratched with a plastic tip. After treatment, the migration was followed at 37 °C for 24 h and photographed at the beginning and end of experiment using a Live Cell Imaging System (Olympus IX2-VCB, Tokyo) at ×40 magnification. The migration distance was calculated using ImageJ software.

### Transwell assay

NMuMG cells were treated with TGF-β1 for 36 h, 2 × 10^5^ cells in the 0.2 % FBS medium were plated into the upper chamber with 8-μm pores (Corning; Life Sciences, MA, USA), and DMEM containing 10 % FBS was placed in the lower chamber. Cells were treated with metformin (10 mM) in the upper chamber and TGF-β1 (5 ng/ml) in the lower chamber. After 24 h, the filters were fixed in methanol for 10 min and stained with 0.1 % crystal violet (Solabri, Beijing) for 12 min. Randomly selected fields were photographed under a microscope, and stained cells were statistically analyzed.

### Statistical analysis

Data are presented as mean ± SD. Significance of differences between groups was tested using Student’s *t* test (*p* < 0.05).

## Results

### LKB1/AMPK inhibits Smad phosphorylation induced by TGF-β

**Fig. 1:**
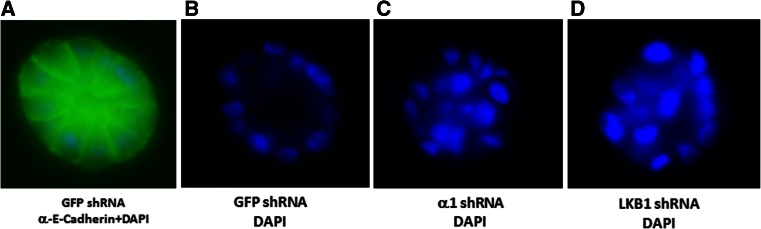
LKB1 and AMPK regulate cell viability. MCF10A cells were infected with lentivirus expressing shRNAs for LKB1, AMPKα1, or GFP. The cells with shRNAs were overlayed on Matrigel bed and cultured for 2 weeks. The mammary cell clusters on the culture chamber were fixed and stained with anti-E-cadherin antibody followed by FITC-conjugated second antibody and DAPI staining. Photos were taken under a fluorescent microscope at ×20 magnification. Representative images are presented. **a** Superimposed image of FITC and DAPI staining. **b** DAPI staining of the acinus derived from GFP shRNA cells. **c** DAPI staining of α1 shRNA cells. **d** DAPI staining of LKB1 shRNA cells

**Fig. 2:**
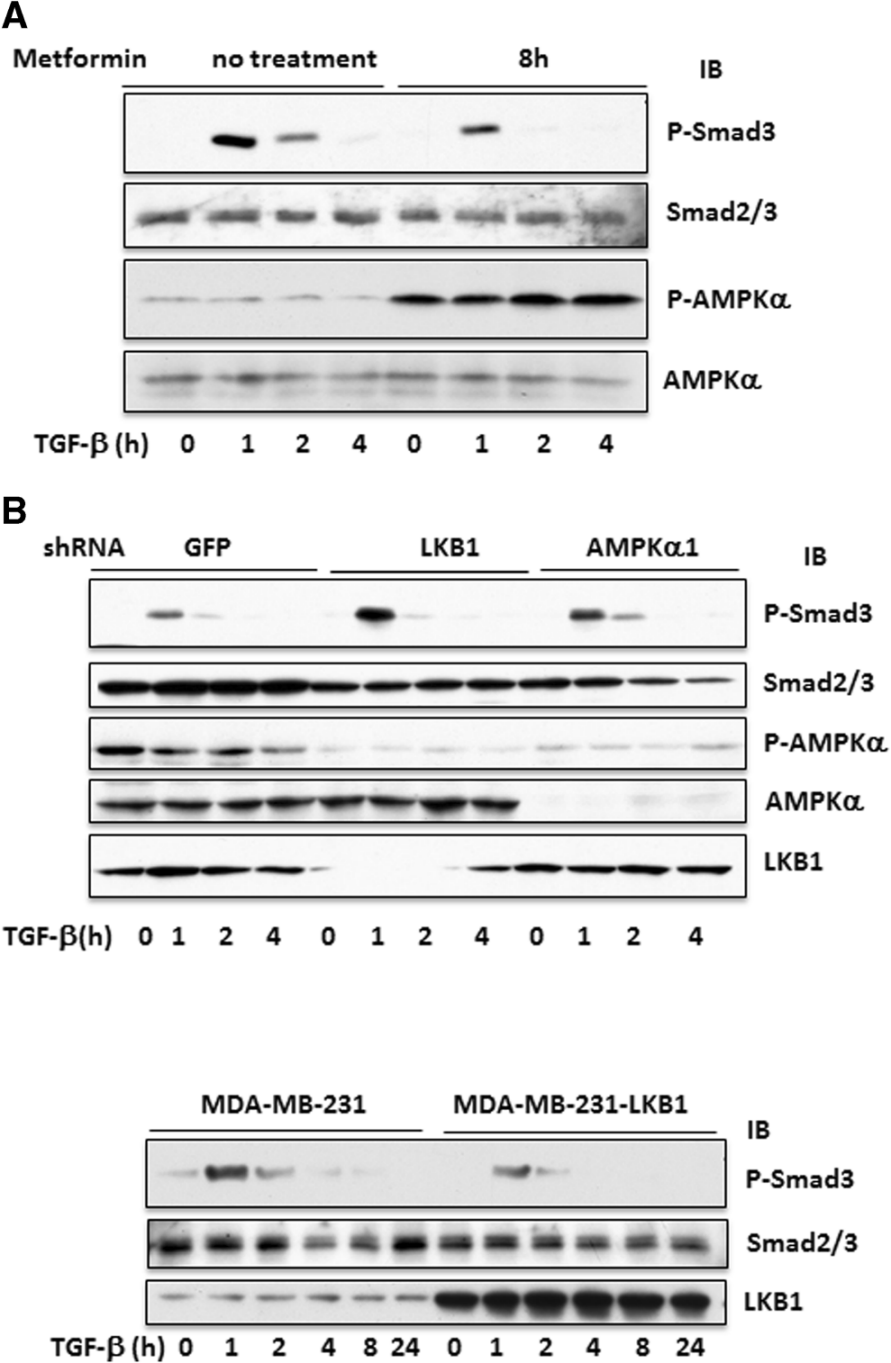
LKB1 inhibits TGF-β-induced Smad2/3 phosphorylation. **a** MCF10A cells were treated with or without metformin (5 mM) for 8 h, followed by TGF-β1 for indicated time. **b** MCF10A cells containing different shRNAs were treated with TGF-β1 for indicated time. **c** MDA-MB-231 cells stably transfected with LKB1 or empty vector as a control were treated with TGF-β1 (5 ng/ml) for different time periods. Equal amounts (20 μg) of cell extracts were immunoblotted with antibodies, as indicated

To examine if LKB1 and AMPK inhibit the function of TGF-β in breast cancerous or precancerous cells, we made stable cell lines by silencing AMPKα1 and LKB1 by shRNA in MCF10A cells and transfecting LKB1 into MDA-MB-231 cells. We first assessed if silencing AMPK and LKB1 promoted cell proliferation or decreased apoptosis of MCF10A cells, an immortalized human mammary epithelial cell line. Our results showed that the doubling time was decreased (data not shown). We then utilized Matrigel to observe acinus formation and compared the ability of the cells bearing GFP shRNA, LKB1 shRNA, and α1 shRNA to form an acinar structure, a parameter of mammary epithelial cell transformation. As shown in Fig. 1, 2 weeks after plating on Matrigel, MCF10A cells formed acinus while the cells with LKB1 or α1 subunit of AMPK silenced failed to do so, suggesting that LKB1/AMPK could inhibit the transformation of mammary epithelial cells. Unfortunately, we could not show the effect of metformin and TGF-β in this setting, as both inhibited the growth of MCF10A cells on Matrigel, where cell clumps became very small. Next, we assessed the effects of metformin on TGF-β-induced Smad phosphorylation in cultured cells. First, our data showed that metformin suppressed the phosphorylation of Smad2/3 induced by TGF-β (Fig. 2a). Second, knockdown of LKB1 and AMPKα1 made the cells more sensitive to TGF-β (Fig. 2b). In contrast, overexpression of LKB1 in MDA-MB-231 cells blunted the Smad2/3 phosphorylation (Fig. 2c).

**Fig. 3:**
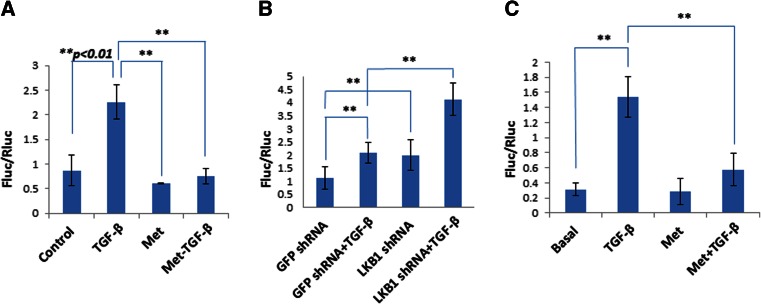
Inhibition of Smad2/3 transcriptional activity by LKB1/AMPK. The TGF-β reporter plasmid encoding firefly luciferase driven by Smad-responsive elements was co-transfected with Renilla luciferase into MCF10A cells (**a**) or into MCF10A cells containing GFP or LKB1 shRNA (**b**). The cells were treated with TGF-β1 (*TGF-β*, 5 ng/ml) and/or metformin (*Met*, 5 mM) for 8 h. Luciferase activity was assayed, and the ratio of two luciferase activities is presented. Similar assays were carried out in MDA-MB-231 cells (**c**). Statistical analysis was performed using Student’s *t* test, and *p* values between groups are indicated

To ascertain if the reduction of Smad phosphorylation resulted in an inhibition of their transcription activity, we conducted luciferase reporter assays. Thus, we transfected plasmid containing firefly luciferase regulated by Smad2/3 response elements and then assessed the responses of MCF10A cells and MDA-MB-231 cells to metformin and TGF-β. As shown in Fig. 3, TGF-β stimulated the promoter activity (Fig. 3a, *p* < 0.01), which was offset by co-treatment with metformin (Fig. 3a, *p* < 0.01). A similar result was obtained with MDA-MB-231 cells (Fig. 3c). When MCF10A cells with LKB1 silenced were used, the luciferase activity was found as high as that in control cells treated with TGF-β (Fig. 3b, *p* < 0.01) and further increased by TGF-β (Fig. 3b, *p* < 0.01). Collectively, these results clearly demonstrated that metformin and LKB1 inhibited the effect of TGF-β on phosphorylation of Smad2/3, leading to suppression of its transcription activity in breast cancer cells and in precancerous lesion.

### Metformin suppresses production of TGF-β1

**Fig. 4:**
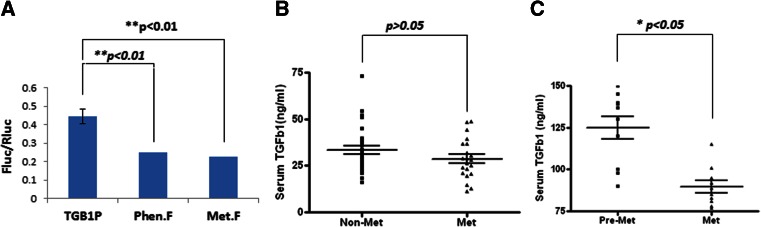
AMPK regulates TGF-β1 production. **a** The promoter region (1.4 kb) was cloned upstream of firefly luciferase in pGL3 vector (*TGB1P*) and the plasmid co-transfected with Renilla luciferase into MDA-MB-231 cells. The cells were treated with metformin (*Met.F*, 5 mM) or phenformin (*Phen.F*, 1 mM) for 8 h. The luciferase assay was performed as in Fig. 3. **b** Serum TGF-β1 levels from type 2 diabetic patients receiving metformin (*Met*) (*n* = 21) or non-metformin (*Non-Met*) (*n* = 29) were measured using an ELISA kit. **c** Serum TGF-β1 from mice (*n* = 10) before (*Pre-Met*) and after Met injection (i.p. 100 mg/kg/day) for 1 week was assayed. The mean with SEM was presented as *horizontal lines*. Significance was tested using Student’s *t* test. **a** **p < 0.01 (mean ± SD, *n* = 5); **b** *p* > 0.05; **c** *p < 0.05

We were curious at the regulation of TGF-β1 expression by metformin. Thus, we cloned genomic DNA encompassing 1.4 kb that covers an essential promoter sequence of TGF-β1 and placed this sequence upstream of the luciferase gene. When this plasmid was transfected into MDA-MB-231 cells and treated with metformin or phenformin, the promoter activity was reduced by about 50 % (*p* < 0.01) (Fig. 4a). We then went on investigating the effect of metformin on serum levels of TGF-β1. First, we collected the sera of patients with type 2 diabetes from the outpatient clinic. Among them, one group was treated only with metformin and another age-matched group was treated with other glucose-lowering drugs but without metformin. We assayed TGF-β1 levels and found that patients with metformin treatment displayed reduced serum concentration of TGF-β1 but this change was not significant (*p* > 0.05), probably due to an insufficient number of patients (Fig. 4b). Next, we carried out the assay in mice, where the sera were collected before and after treatment with metformin for 1 week and TGF-β1 was measured. The result revealed that serum levels of TGF-β1 were significantly reduced (*p* < 0.05) (Fig. 4c). Thus, our results revealed a new role of AMPK in TGF-β1 production.

### Metformin inhibits mouse EMT induced by TGF-β

**Fig. 5:**
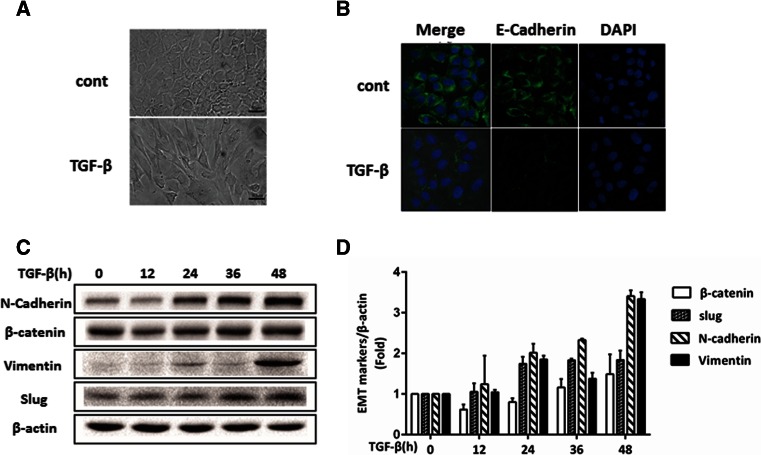
Induction of EMT by TGF-β. NMuMG cells were incubated with TGF-β1 (5 ng/ml) up to 48 h. The morphology of EMT was examined under a light contrast microscope (**a**), by indirect immunofluorescent labeling of E-cadherin (FITC labeled) and DAPI staining of the nucleus (**b**) and by Western blotting with antibodies against EMT (**c**). Scan densitometry was used to determine Western blot signals of **c** from three independent experiments and plotted in **d**

In this study, we attempted to use mouse NMuMG cells, as they appeared to be a better cell model in our hands. When NMuMG cells were treated with TGF-β1 for 48 h, morphological changes toward mesenchymal cells were easily achieved, accompanied with a decrease in E-cadherin and increases in N-cadherin, β-catenin, vimentin, and Slug (Fig. 5).

**Fig. 6:**
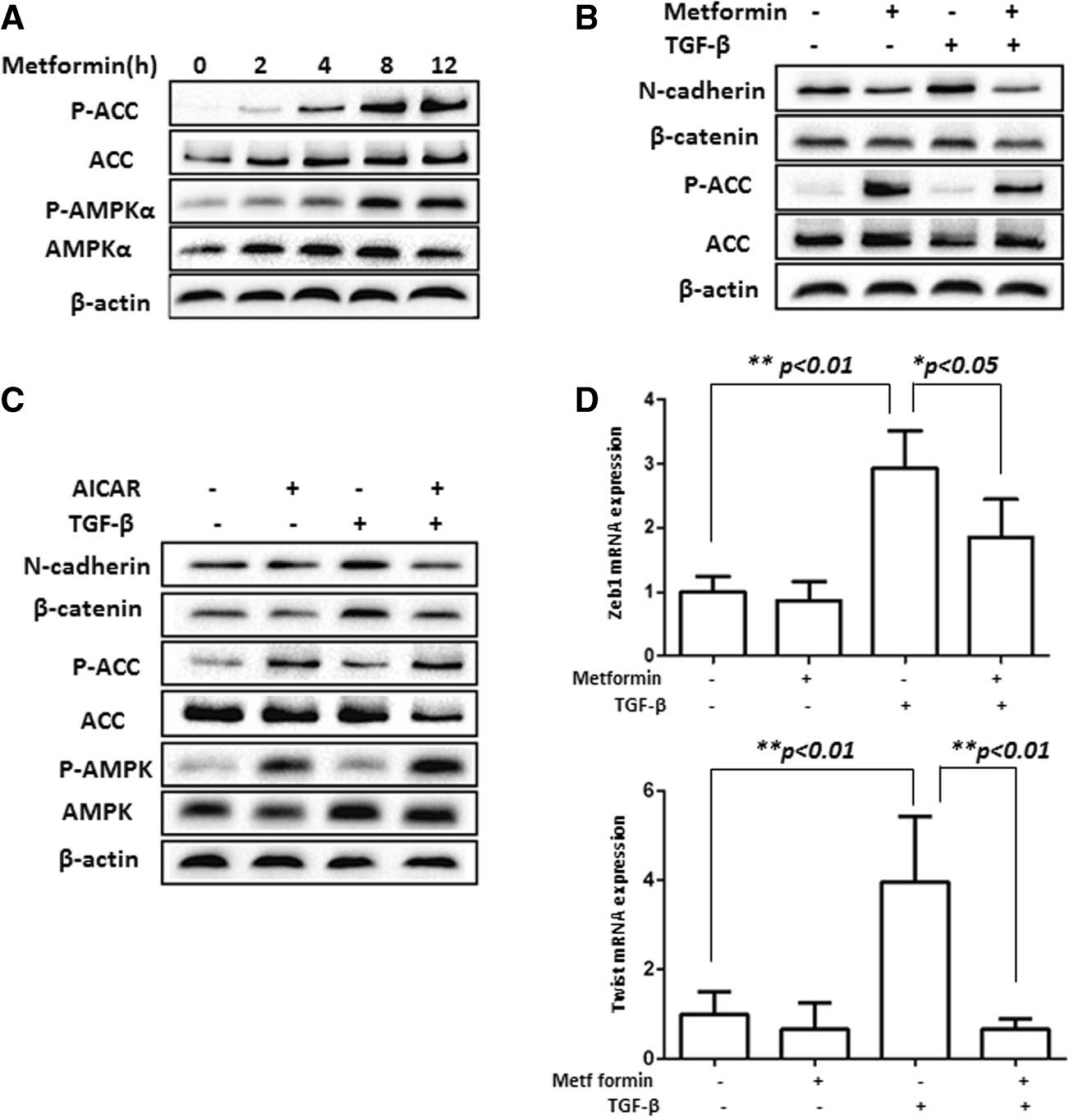
Metformin suppresses induction of EMT markers in NMuMG cells by TGF-β. **a** NMuMG cells were treated with metformin (10 mM) for different time periods up to 12 h. **b** The cells were treated with or without AICAR (1 mM) ± TGF-β for 48 h. **c** The cells were treated with or without metformin ± TGF-β for 48 h. **a**–**c** Equal amounts of cell extracts were immunoblotted with antibodies, as indicated. **d** The cells were treated as in **c**, total RNA was extracted, and real-time PCR was performed on ZEB1 and Twist. The *graph* represents averages normalized with GAPDH in a triplicate experiment (mean ± SD, *n* = 3). Statistical analysis was performed using Student’s *t* test, and *p* values between groups are indicated

**Fig. 7:**
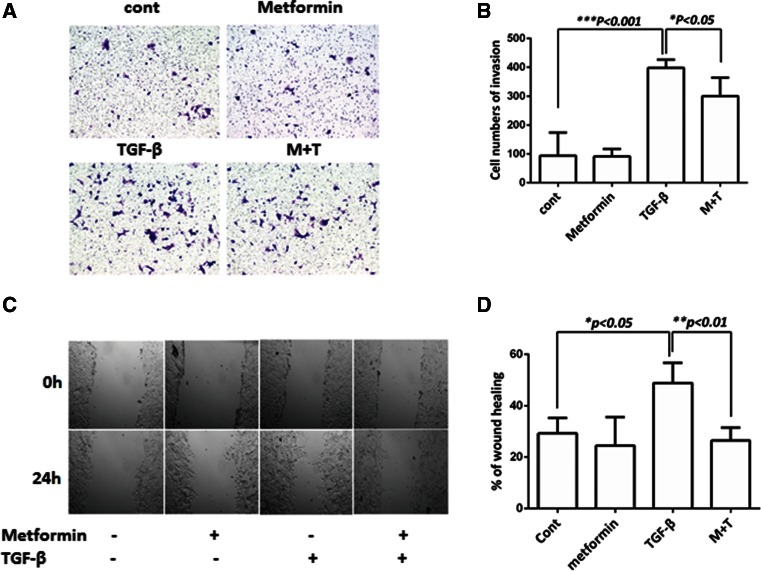
Metformin inhibits the ability of TGF-β to stimulate the migration of NMuMG cells. **a** Migration through transwell membranes. NMuMG cells (30,000/wells) were plated on the top of membranes, and the cells were allowed to migrate for 24 h under the treatment, as indicated. The migrated cells were stained with crystal violet, and the randomly selected areas were photographed (*left*). Five randomly selected spots were counted and plotted (*right*) with control as 100 %. **b** Wound healing analysis. NMuMG cells were grown to confluence, scratched with a pipette tip, and cultured for 24 h. The scratched areas were photographed at 0 and 24 h. The migration rate was calculated with ImageJ software. The *graph* represents values from three independent experiments (mean ± SD). Statistical analysis was performed using Student’s *t* test, and p values between groups are indicated. *Cont* control, *M + T* metformin + TGF-β1

To assess the effect of metformin on EMT, we tested the ability of metformin to activate AMPK in NMuMG cells and found that AMPK was activated well by metformin (Fig. 6a). Then, we treated the cells with TGF-β1 alone or together with metformin or 5-amino-4-imidazolecarboxamide riboside (AICAR) for 48 h. Our results showed that the stimulatory effects of TGF-β1 on mesenchymal markers were abrogated by metformin or AICAR (Fig. 6b–d). Since EMT often acquires accelerated cell migration, we assessed if the stimulation of cell migration by TGF-β1 was suppressed by metformin. Our data revealed that TGF-β stimulated the migration of NMuMG cells on transwell and would healing assays, which were significantly inhibited by metformin (Fig. 7). Taken together, our results showed that TGF-β stimulated EMT and migration of NMuMG cells, all of which were compromised by AMPK activation (i.e., metformin or AICAR), underpinning an inhibitory role of AMPK.

## Discussion

TGF-β plays a tumor-suppressing role in early stages of tumorigenesis but a promoting role in tumor progression in late stages. Thus, activation of this signaling pathway delays the growth of primary tumor but enhances tumor metastasis [30]. In recent years, regulation of TGF-β signaling by AMPK has emerged as an important topic in cancer biology because of their opposite roles in cancer metastasis, EMT, and fibrosis [18–25]. In the present study, we have shown that LKB1/AMPK possesses an inhibitory role in TGF-β signaling. Thus, the presence or overexpression of LKB1 diminished the responses of breast cancer cells and precancerous mammary epithelial cells to TGF-β, leading to reduced phosphorylation of Smad2/3 and its transcription activity, whereas knockdown of LKB1 and AMPKα1 resulted in opposite changes and disruption of the acinus structure derived from mammary epithelial cells. Interestingly, our results revealed that metformin and phenformin suppressed the promoter activity of TGF-β1. In keeping with this, TGF-β1 levels in the sera of patients with type 2 diabetes who received metformin exhibited a trend of reduction, as compared with those without metformin. Furthermore, they were significantly reduced in the sera of mice that were administered with metformin for a week. Finally, our data showed that activation of AMPK inhibited TGF-β1-mediated EMT. Hence, our study supports that AMPK could be a suitable drug target to correct the pathophysiology of diseases engendered by dysregulated TGF-β.

Three-dimensional culture on Matrigel has been used to assess dysregulated proliferation and reduced apoptosis of mammary epithelial cells [28]. Normal epithelial cells undergo apoptosis in the middle of mammary cell cluster and result in a hollow structure like acinus, while transformed cells are resistant to apoptosis and thus cannot form acinus. In this study, we found that knockdown of LKB1 and AMPK disturbed the acinar structure, suggesting that LKB1/AMPK inhibits cell growth and/or induces apoptosis in the area of crowded cell clumps, which is in line with our previous finding [31].

It has been shown that AMPK activated by a variety of maneuvers, such as pharmacological activators or activating mutation, inhibits TGF-β -mediated cellular events. Until now, studies have suggested that mainly three mechanisms account for AMPK inhibition of the TGF-β signal transduction pathway: (1) Smad3-dependent, but phosphorylation-independent [27] event; (2) Smad3 phosphorylation-dependent [26] event; and (3) p300-dependent event. In the third regulation, AMPK induces degradation of p300, leading to decreased acetylation of Smad3 and disruption of the complex between p300 and Smad3 [19]. Our present study showed that AMPK suppressed TGF-β-induced phosphorylation of Smad2/3, thereby inhibiting its transcription activity. Furthermore, our study adds an additional aspect of regulation in which AMPK suppresses the transcription and production of TGF-β1.

Three TGF-β isoforms exist, β1, β2 and β3, which are encoded by distinct genes and exhibit different expression patterns and physiological and pathophysiological activities in different cells and systems [32, 33]. The expression of TGF-β is controlled by an auto-feed-back loop. The promoter regions of TGF-β1 and TGF-β3 contain a Smad response element such that the transcription is auto-regulated [32, 33]. In our study, we found that TGF-β1 was regulated by AMPK at the transcription level but it is not clear if this occurs through inhibition of Smad2/3 phosphorylation or an independent event. In any case, it is an important finding in view of the fact that TGF-β1 is upregulated in tissue fibrosis and advanced cancer.

A prior report showed that the serum level of TGF-β1 was higher in type 2 diabetes than normal population but did not find an effect of metformin [34]. A second study by measuring serum levels of TGF-β1 in type 1 diabetes revealed that TGF-β1 was elevated in the patients with retinopathy, suggesting that it is involved in complications and can serve as a progression marker for the disease [35]. Many studies have reported elevation of TGF-β in advanced cancer [8–11]. In our current study, we observed a decrease in the serum concentration of TGF-β1 in patients with type 2 diabetes treated with metformin, as compared to non-metformin group, but it was not significant. We are aware that our comparison may need to be modified, as an appropriate comparison would be made before and after treatment metformin. However, it is difficult to conduct this sort of study, as TGF-β expression levels may vary upon the disease stage and diabetic symptoms may not be so severe to allow detection of the increase of TGF-β at the beginning of metformin administration. To overcome this obstacle, we will have to expand the sample size and to carry out prospective studies on diabetic patients treated with and without metformin for years. To rectify the flaws of our human study, we treated mice with metformin for 1 week, collected blood prior to and post treatment, and measured serum TGF-β1 levels. Our results showed a significant reduction after treatment. Of note, the values of TGF-β1 levels in our study were somewhat different from previously published data [34–36]. This was probably caused by the use of different methods. For example, some of these studies measured total TGF-β (i.e., acidification of serum allows measurement of bound and free TGF-β) while others assayed free TGF-β. In the present study, we used a kit to measure total serum TGF-β1.

In summary, we have shown that AMPK by activation of metformin, AICAR, or LKB1 exerts a negative effect on TGF-β signaling, leading to inhibition of Smad2/3 activation and suppression of EMT and cell migration in breast cancer cells and precancerous mammary epithelial cells. Interestingly, we have shown that metformin inhibits transcription of TGF-β1 and reduces serum levels of TGF-β1. Our next phase of study will be to decipher the mechanism by which AMPK regulates TGF-β1 transcription. In sum, our study supports that AMPK could serve as a drug target in the treatment of breast cancer progression as well as other disorders which are aggravated by elevated TGF-β1.

*AICAR* 5-amino-4-imidazolecarboxamide riboside, *AMP* adenosine monophosphate, *AMPK*, AMP-activated protein kinase, *CSC* cancer stem cell, *EMT* epithelial-to-mesenchymal transition, *GAPDH* glyceraldehyde 3-phosphate dehydrogenase, *LKB1* liver kinase B1, *SNAIL1/2* transcription repressors for E-cadherin, *TGF-β* transforming growth factor beta, *ZEB1* zinc finger E-box binding homeobox 1
